# Genomics of Blood Pressure and Hypertension: Extending the Mosaic Theory Toward Stratification

**DOI:** 10.1016/j.cjca.2020.03.001

**Published:** 2020-05

**Authors:** Stefanie Lip, Sandosh Padmanabhan

**Affiliations:** Institute of Cardiovascular and Medical Sciences, University of Glasgow, Glasgow, Scotland, United Kingdom

## Abstract

The genetic architecture of blood pressure (BP) now includes more than 30 genes, with rare mutations resulting in inherited forms of hypertension or hypotension, and 1477 common single-nucleotide polymorphisms (SNPs). These signify the heterogeneity of the BP phenotype and support the mosaic theory of hypertension. The majority of monogenic syndromes involve the renin-angiotensin-aldosterone system and the adrenal glucocorticoid pathway, and a smaller fraction are due to rare neuroendocrine tumours of the adrenal glands and the sympathetic and parasympathetic paraganglia. Somatic mutations in genes coding for ion channels (*KCNJ5* and *CACNA1D*) and adenosine triphosphatases (*ATP1A1* and *ATP2B3*) highlight the central role of calcium signalling in autonomous aldosterone production by the adrenal gland. The per-SNP BP effect is small for SNPs according to genome-wide association studies (GWAS), and all of the GWAS-identified BP SNPs explain ∼ 27% of the 30%-50% estimated heritability of BP. Uromodulin is a novel pathway identified by GWAS, and it has now progressed to a genotype-directed clinical trial. The majority of the GWAS-identified BP SNPs show pleiotropic associations, and unravelling those signals and underpinning biological pathways offers potential opportunities for drug repurposing. The GWAS signals are predominantly from Europe-centric studies with other ancestries underrepresented, however, limiting the generalisability of the findings. In this review, we leverage the burgeoning list of polygenic and monogenic variants associated with BP regulation along with phenome-wide studies in the context of the mosaic theory of hypertension, and we explore potential translational aspects that underlie different hypertension subtypes.

## Résumé

L'architecture génétique de la pression artérielle (PA) comprend maintenant plus de 30 gènes, avec des mutations rares entraînant des formes héréditaires d'hypertension ou d'hypotension et 1 477 polymorphismes d’un seul nucléotide (PSN) courants. Tout cela démontre l'hétérogénéité du phénotype de la PA et soutient la théorie « mosaïque » de l’hypertension. La majorité des syndromes liés aux maladies monogéniques impliquent le système rénine-angiotensine-aldostérone et la voie des glucocorticoïdes via les glandes surrénales, tandis qu’une plus petite fraction est due à de rares tumeurs neuroendocrines des glandes surrénales et des paraganglions sympathiques et parasympathiques. Des mutations somatiques dans les gènes codant les canaux ioniques (KCNJ5 et CACNA1D) et les adénosine triphosphatases (ATP1A1 et ATP2B3) mettent en évidence le rôle central de la signalisation du calcium dans la production autonome d'aldostérone par la glande surrénale. L'effet de PSN liés à la PA est faible pour les PSN identifiés dans les études d'association pangénomique (EAP), et tous les PSN de la PA identifiés par EAP expliquent ∼ 27 % des 30 %-50 % d'héritabilité estimée de la PA. L'uromoduline représente une nouvelle voie de signalisation identifiée par les EAP, et elle fait désormais l’objet d’un essai clinique basé sur le génotype. La majorité des PSN liés à la PA identifiés par des EAP présentent des associations pléiotropes, et l'élucidation de ces signaux et des voies biologiques sous-jacentes offre des possibilités potentielles de réorientation de médicaments. Les données des EAP proviennent principalement d'études de populations d'Europe, tandis que les autres ascendances y sont sous-représentées, ce qui limite la généralisation des résultats. Dans cette revue de littérature, nous nous appuyons sur la liste émergente des variants polygéniques et monogéniques associées à la régulation de la PA ainsi que sur des études à l'échelle du phénome dans le contexte de la théorie « mosaïque » de l'hypertension, et nous explorons les aspects translationnels potentiels qui sous-tendent différents sous-types d'hypertension.

Page’s “mosaic theory” of hypertension posited that essential hypertension (HTN) is not a single disease, but several different diseases with different origins and development—all causing HTN and its consequences.^1^ The mosaic of causes of hypertension, if it exists for essential hypertension, needs elucidation because it potentially opens new avenues for stratification, new drug development, and precision medicine. Blood pressure (BP) genomics has been one of the most challenging research areas, primarily because the inherent variability of BP and measurement errors (both human and instrument related) substantially dilute the statistical power of any discovery study. In this review, we leverage the current catalogue of polygenic variants and monogenic variants associated with BP regulation along with phenome-wide studies to determine whether there is evidence for the mosaic theory, and we explore potential translational aspects that underlie different HTN subtypes.

## Monogenic Syndromes

**Figure 1:**
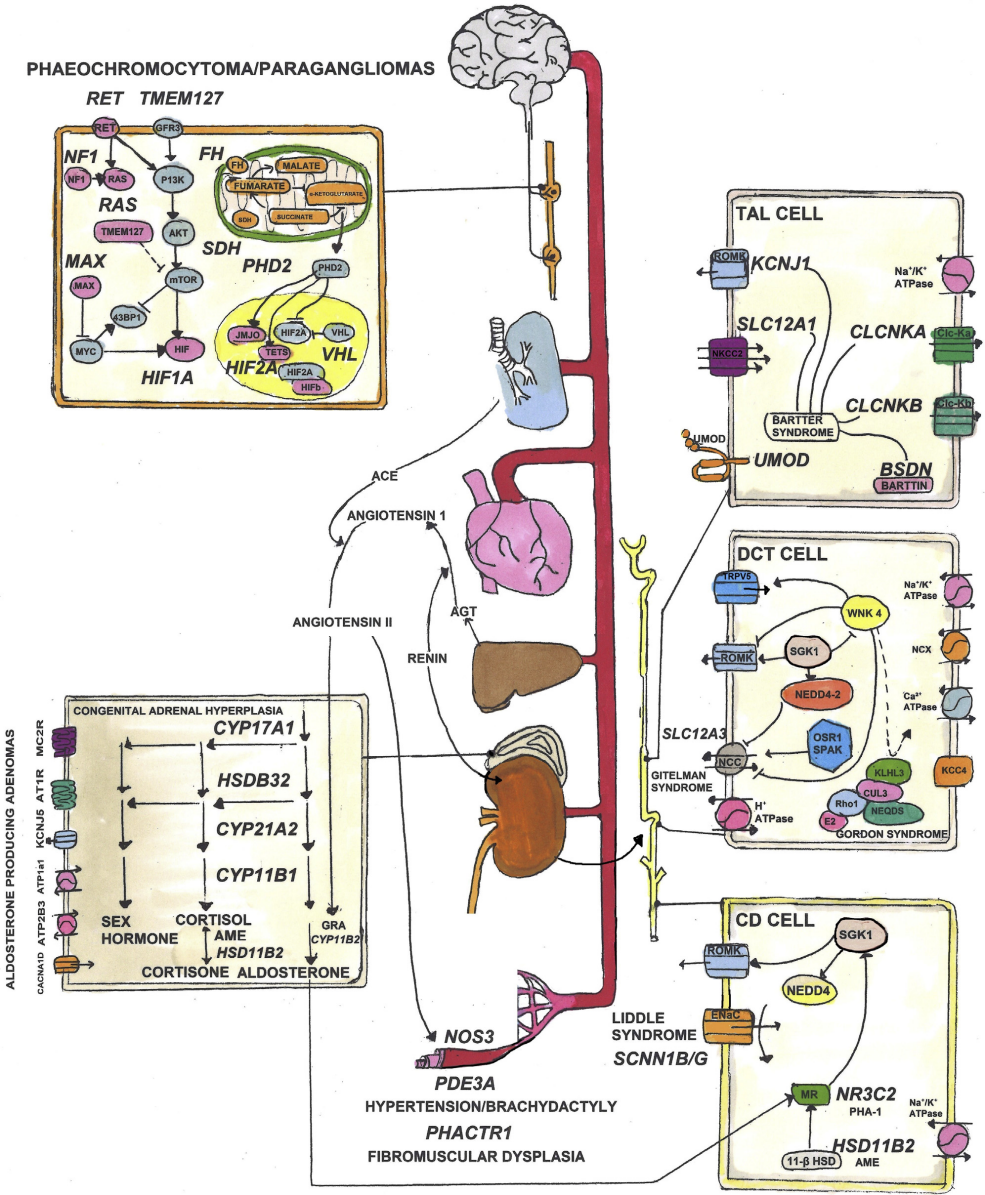
Pathways in the circulatory, endocrine, and neurologic systems that are associated with monogenic forms of hypertension. Causal monogenic genes and their syndromes are described in Table 1.

There is evidence from monogenic forms of HTN that they are caused by specific pathways perturbed by rare causal mutations in single genes resulting in an early and severe BP phenotype.^2^ The monogenic forms are the most successful examples of gene mapping, with mutations in more than 31 genes now linked to perturbed gene function and BP dysregulation, enhancing our understanding of both the mechanisms and the treatment of HTN. Molecular and clinical details of monogenic syndromes have been reviewed in detail recently, and we refer to the reader to these articles.^2^^,^^3^ Table 1 and Figure 1 summarise the monogenic syndromes, their causal genes, mechanisms, and management. Different monogenic syndromes all present with the same BP phenotype but are essentially separate diseases differentiated by additional clinical or laboratory characteristics and the causative genetic mutation. Although monogenic forms of HTN account for a small fraction of the public health burden of hypertension, studies of monogenic syndromes have established the genetic basis for the “known knowns” of BP regulation. These are major pathways of BP regulation, centred on sodium balance and the renin-angiotensin-aldosterone system, originally inferred from physiological studies that led to the development of almost all of the commonly used antihypertensive drugs.^4^ For example, one of the most effective drugs for HTN is spironolactone, which antagonises the effect of aldosterone. It was discovered in 1957 and has been used in clinical practice since 1959. The first case of familial primary aldosteronism was described in 1966 with the discovery of the causal chimeric mutation by Lifton et al.^5^ in 1992, 25 years later. Spironolactone, was recently shown to be overwhelmingly the most effective drug treatment for resistant HTN in the PATHWAY-2 randomised controlled trial.^6^ Interestingly, somatic gain-of-function mutations in (*KCNJ5)* account for 40% of patients with an aldosterone-producing adenoma, whereas 7% are due to mutations in the α-subunit of Na^+^-K^+^-adenosine triphosphatase (ATPase; *ATP1A1*), plasma membrane Ca^2+^-ATPase (*ATP2B3*), and L-type Ca^2+^ channel CaV1.3 (*CACNA1D*).^3^ Mutations in these genes are less frequent in inherited cases of primary hyperaldosteronism and raises the possibility that HTN could be due to a multiplicity of uncommon variants.^3^ The majority of monogenic syndromes involve the renin-angiotensin-aldosterone system and the adrenal glucocorticoid pathway with treatment directed toward ultimately reducing aldosterone and angiotensin and increasing Na^+^ excretion (Table 1). A smaller fraction of monogenic HTN syndromes are due to rare neuroendocrine tumours of the adrenal glands and the sympathetic and parasympathetic paraganglia: pheochromocytomas (PCCs) and paragangliomas (Table 1). Autosomal hypertension with type E brachydactyly is not related to salt reabsorption but due to mutation in the *PDE3A* gene resulting in enhanced activity of PDE3A leading to increased neointimal proliferation and remodelling of the arteries and neurovascular structures.^7^

**Table 1:** Monogenic syndromes of blood pressure dysregulation with causal genes, key features and treatment

Syndrome	Gene	Mechanism	Key features	Treatment
11β-Hydroxylase deficiency	*CYP11B1*	Group of autosomal recessive disorders that impair cortisol biogenesis with consequent overproduction of corticotropin-releasing hormone and ACTH and adrenal gland hyperplasia. This leads to either hypotension or hypertension.	Neonatal onset. Virilisation, short stature, suppressed aldosterone and renin.	Glucocorticoid therapy
17a-Hydroxylase deficiency	*CYP17A1*		Hypertension, hypokalemic alkalosis. Increased ACTH and follicle-stimulating hormone. Absent sexual maturation.	Glucocorticoid therapy, potassium-sparing diuretics
21-Hydroxylase deficiency	*CYP21A2*		Short stature, decreased fertility, hirsutism. Salt wasting associated with poor feeding, weight loss, dehydration, and vomiting in babies.	Glucocorticoid therapy
3β-Hydroxysteroid dehydrogenase	*HSD3B2*		Primary hypoadrenalism with virilisation in females and undervirilisation in males. Severe form presents in infancy with salt wasting and adrenal crisis.	Glucocorticoid therapy
Apparent mineralocorticoid excess (AME)	*HSD11B2*	11β-Hydroxysteroid dehydrogenase (HSD11B2) activity is reduced or absent. This results in failure of cortisol conversion to cortisone, leading to inappropriate activation of the MR by cortisol and hypertension.	Increased plasma ACTH. Increased urinary cortisol-cortisone ratio. Low plasma renin and aldosterone.	Low-sodium diet and spironolactone
Bartter syndrome	*CLCNKA* *CLCNKB* *KCNJ1* *MAGED2* *SLC12A1*	Mutations in the *SLC12A1* gene cause type I, *KCNJ1* type II, *CLCNKB* type III, and *BSND* or a combination of mutations in the *CLCNKA* and *CLCNKB* genes type IV. Bartter syndrome results from defective salt reabsorption in the thick ascending limb of loop of Henle.	Low blood pressure. Impaired chloride reabsorption in the thick ascending loop of Henle leads to impaired sodium reabsorption. Hypokalemic metabolic alkalosis. Increased plasma renin and aldosterone.	Potassium supplementation and use of cyclooxygenase inhibitors, angiotensin-converting enzyme inhibitors, and potassium-sparing diuretics.
Familial hyperaldosteronism (FH I)	*CYP11B1* *CYP11B2*	Hypertension caused by ACTH-driven aldosterone secretion. A chimeric fusion protein with the 5′ regulatory sequences of 11β-hydroxylase (*CYP11B1*) (which confers ACTH responsiveness) and the 3′ coding sequence of aldosterone synthase (*CYP11B2*) results in ACTH becoming the main controller for aldosterone secretion instead of angiotensin II or potassium.	Plasma and urinary aldosterone responsive to ACTH; dexamethasone suppressible within 48 hours. Increased aldosterone and low renin.	Dexamethasone
Familial hyperaldosteronism (FH II)	Linkage to Chr 7p22, *KCNJ5*	Unknown mechanism. Missense mutations in *KCNJ5* have been identified in rare cases.	May present either as an APA, bilateral adrenal hyperaldosteronism (BAH), or both. Fatigue, and muscle weakness. Hypokalemia seen in 25% of patients.	Adrenalectomy is performed in case of APA, medical therapy with aldosterone antagonists in case of BAH.
Gitelman syndrome	*SLC12A3* *CLCNKB*	Loss-of-function mutations in either NCC encoded by *SLC12A3* or *CLCNKB* lead to decreased NaCl reabsorption in the distal convoluted tubule, resulting in hypovolemia and activation of the renin-angiotensin-aldosterone system.	Low blood pressure. Increased plasma renin. Renal potassium and magnesium wasting.	Oral potassium and magnesium supplementation with adequate salt and water.
Hypertension and brachydactyly syndrome	*PDE3A*	Mutations in *PDE3A* increase protein kinase A–mediated PDE3A phosphorylation, increased cAMP hydrolysis and lowered cAMP levels. This results in a gain of function in PDE3A activity. The increase in cAMP hydrolysis causes reduced levels of cAMP levels in vascular smooth muscle cells which, in turn, increases neointimal proliferation and remodelling of the arteries and neurovascular structures.	Brachydactyly type E, short phalanges, short metacarpals	Possible role for PDE3 inhibition
Hypertension exacerbation in pregnancy	*NR3C2*	Heterozygous mutation of the MR leads to altered nuclear receptor ligand selectivity and activation. Steroid hormones, such as progesterone, have increased affinity for the MR, leading to enhanced activation of mineralocorticoid signalling cascades (increases in ENaC and Na/K–adenosine triphosphatase activity) that increase Na^+^ reabsorption and K^+^ secretion.	Low renin, low aldosterone, hypokalemia. Progesterone and other steroids lacking 21-hydroxyl groups, normally MR antagonists, becoming potent agonists.	Spironolactone contraindicated; sodium chloride treatment. Delivery of the foetus ameliorates hypertension.
Liddle syndrome	*SCNN1B* *SCNN1G*	Autosomal dominant. Caused by heterozygous mutations in *SCNN1B* or *SCNN1G* that results in truncated C-terminus on either the beta or gamma subunits of ENaC, removing a binding site for NEDD-4. This results in Constitutive activation of ENaC.	Salt-sensitive hypertension that develops early in childhood. Low plasma renin and aldosterone. Hypokalemia.	Low sodium diet. Amiloride or triamterence.
Multiple endocrine neoplasia, type IIA (MEN2 syndrome)	*RET*	Gain-of-function mutations of *RET* causes MEN2 syndrome because normal development of the kidneys and the sympathetic, parasympathetic, and enteric nervous system is dependent on *RET*.	Associated with multiple endocrine neoplasms, including medullary thyroid carcinoma, pheochromocytoma, and parathyroid adenomas	Alpha adrenergic blockers for pheochromocytoma
Paragangliomas (PGL1-5)	*SDHA* *SDHAF2* *SDHB* *SDHC* *SDHD*	SDH is a mitochondrial enzyme complex consisting of four subunits: SDHA, SDHB, SDHC, and SDHD involved in the tricarboxylic acid cycle. The *SDHx* genes are thought to function as classical tumour suppressors, and mutations in any of the *SDHx* genes abolishes SDH enzyme activity and protein expression.	Multiple catecholamine-secreting paragangliomas and pheochromocytomas	Surgery, adrenergic blockers (alpha-blockade followed by beta-blockade)
Pseudohypoaldosteronism (PHA II; Gordon syndrome)	*CUL3* *KLHL3* *NR3C2* *WNK1* *WNK4*	Autosomal dominant. Mutant WNK1 results in activation of SPAK, leading to enhanced phosphorylation of NCC, increased NaCl reabsorption and hypertension. Overexpression of WNK1 can inhibit WNK4 activity, further promoting additional NCC phosphorylation and NaCl reabsorption. Mutations in WNK4 disrupts its binding to KLHL3, leading to increased levels of WNK4 and hypertension. CUL3 and KLHL3 mutations disrupt proteolytic degradation of the WNKs leading to increased levels of WNK4.	Hypertension, hyperkalemia, hyperchloremic metabolic acidosis	Thiazide diuretics, prostaglandin inhibitors, alkalising agents, and potassium-binding resins. Na^+^- and K^+^-restricted diet.
Sporadic aldosterone-producing adenoma (APA), or primary aldosteronism	*ATP1A1* *ATP2B3* *CACNA1D* *KCNJ5*	Somatic gain-of function mutations in the inward rectifier potassium channel KCNJ5 (Kir3.4) is present in ∼ 40% of APAs. The mutations increase channel sodium permeability, leading to increased calcium influx through voltage-gated calcium channels. This stimulates aldosterone secretion and cell proliferation and APA development. A similar mechanism underlies somatic mutations in *CACNA1D*, *ATP1A1*, and *ATP2B3*.	Hyperaldosteronism, hypertension, hypokalemia	Surgery, aldosterone antagonists
von Hippel–Lindau syndrome	*VHL*	Germline mutations that inactivate the *VHL* gene possibly interfere with oxygen-dependent regulation of hypoxia-inducible factor.	Associated with retinal, cerebellar, and spinal hemangioblastoma, renal cell carcinoma, pheochromocytoma, and pancreatic tumours	

ACTH, adrenocorticotropic hormone; cAMP, cyclic adenosine monophosphate; ENaC, epithelial sodium channel; MR, mineralocorticoid receptor; NCC, sodium-chloride cotransporter.

Although the rare monogenic syndrome may not have significant direct public health impact, the indirect global influence of drugs targeting those specific pathways among those with essential HTN is substantial. It is in this context that the potential value of the global efforts to discover the genetic basis of essential or polygenic HTN needs to be considered. Thus, monogenic syndromes fulfil Page’s mosaic theory of HTN, albeit in a smaller subset of all hypertensive individuals.

## Essential Hypertension

It logically follows that essential HTN may be a *forme fruste* of monogenic HTN, with minor variations in the monogenic genes leading to milder and later-onset HTN. Consequently, they may exhibit an underlying mosaic pattern. There are indications that this may be a possibility—for example, individuals of African ancestry tend to have a salt-sensitive form of HTN,^8^ in contrast to individuals of European ancestry; somatic mutations causing hyperaldosteronism^9^ result in another subset of HTN. However, beyond these 2 examples, it remains to be established if essential HTN is a scaled-up mosaic form of monogenic HTN.

Beyond the evidence from monogenic forms of HTN, there are multiple lines of observational evidence suggesting that BP has a genetic component. 1) Family and twin studies have established that BP heritability ranges from 15% to 40% for clinic systolic blood pressure (SBP), and from 15% to 30% for clinic diastolic blood pressure (DBP); for ambulatory BP (sleep), heritability was 69% and 51% for SBP and DBP, respectively.^10^^,^^11^ 2) The risk of developing HTN is significantly increased in individuals with 1 or 2 hypertensive parents,^12^ and monozygotic twins show higher BP correlations than dizygotic twins.^13^ And 3) in the burst of discovery in the genome-wide era, the search for common genetic variants underlying BP is based on genome-wide association studies (GWAS) which are hypothesis-generating studies that scan the entire genome to find associations between genetic variants (typically single-nucleotide polymorphisms [SNPs]) and a phenotype. The first GWAS of HTN was entirely negative, leading many to question if BP and HTN was genetically tractable or if its genetic component was trivial.^14^ However, since 2007, a series of sequential GWAS of BP and HTN with sample sizes exponentially increasing to the latest study involving 1 million subjects have identified more than 1477 SNPs associated with BP traits explaining about 27% of the 30%-50% estimated heritability of BP.^2^^,^^15^ All of these support a multifactorial polygenic basis for BP regulation and HTN—the Pickering argument from the 1950s Platt-Pickering debate.^16^

### Novel pathways from GWAS

Despite the plethora of common variants arising from GWAS, the biggest challenge has been linking these variants to a causal mechanism in the BP regulatory pathway. The main reason for this is because GWAS SNPs are selected for screening the genome based on linkage-disequilibrium patterns, and this results in the majority of the signals being in noncoding or intergenic regions. Nevertheless, there are examples of the value of GWAS in identification of novel pathways of BP regulation point to potential subgroups with common underlying pathways that may offer avenues for targeted screening or therapy. We start with 2 SNPs (near *UMOD* and *PHACTR1/EDN1* genes) that have identified novel pathways with early translational potential because they involve gene products that are the targets for licensed drugs. Then we look at pharmacogenetic interactions that validate pathways targeted by current antihypertensive drugs and explore opportunities for repurposing drugs or tailoring treatment.

#### Uromodulin

A GWAS of BP extremes^17^ identified a 5′-promoter SNP, rs13333226, near the uromodulin gene (*UMOD*) which is associated with BP and uromodulin excretion. *UMOD* is almost exclusively expressed in the thick ascending limb of the loop of Henle in the kidney, where 25% of the filtered Na^+^ is reabsorbed, pointing to a novel sodium-based BP pathway. A potential interaction between uromodulin and the main sodium transporter, NKCC2, in the thick ascending limb of the loop of Henle was established with transgenic mice experiments.^18^^,^^19^ In *Umod-*knockout mice, NKCC2 shows reduced cotransporter activity with consequent greater sodium excretion and a 20 mm Hg lower BP compared with wild-type mice.^18^ The role of NKCC2 was further established by Trudu et al.,^19^ who showed in both mice overexpressing *Umod* and hypertensive individuals homozygous for the *UMOD* increasing allele that the NKCC2 antagonist furosemide had a greater natriuretic and hypotensive effect. This result is currently being tested in a clinical trial (ClinicalTrials.gov Identifier: NCT03354897) to reposition loop diuretics in the HTN care pathway.

#### *PHACTR1*

An intronic SNP in the phosphatase and actin regulatory protein 1 (*PHACTR1*) gene associated with increased risk of coronary artery disease (CAD) and coronary calcification and decreased risk of migraine headache, cervical artery dissection, fibromuscular dysplasia, and HTN.^20^^,^^21^ Functional analysis of this variant indicated that it is a distal regulator of endothelin (ET) 1 (*EDN1*), a gene located 600 kb upstream of *PHACTR1*.^20^ Thus, this functional SNP may potentially be associated with a lifetime’s exposure of at least 20% higher ET-1 precursor plasma levels, but this requires more validation.^20^^,^^22^ *EDN1* causes vasoconstriction and cell proliferation through activation of ET_A_ receptors (*EDNRA*) on vascular smooth muscle cells and vasodilatation via release of nitric oxide and prostacyclin (*PGI2*) through activation of ET_B_ receptors (*EDNRB*). Endothelin receptor antagonists show BP-lowering properties with both ET_A_-selective and nonselective drugs (bosentan, darusentan).^23^^,^^24^ However, ET receptor antagonists did not gain traction as antihypertensive agents because bosentan resulted in liver dysfunction and fluid retention and darusentan did not meet its prespecified coprimary end points. A new phase III trial, the PRECISION study (ClinicalTrials.gov Identifier: NCT02603809) is currently underway to test the efficacy of aprocitentan in resistant hypertension.^23^^,^^24^ Endothelin receptor antagonists are now licensed for the treatment of pulmonary HTN.^24^ In addition, a genotype-directed use of endothelin antagonists for nonobstructive CAD may offer a solution for targeted treatment (ClinicalTrials.gov Identifier: NCT04097314).

## Pleiotropy

**Figure 2:**
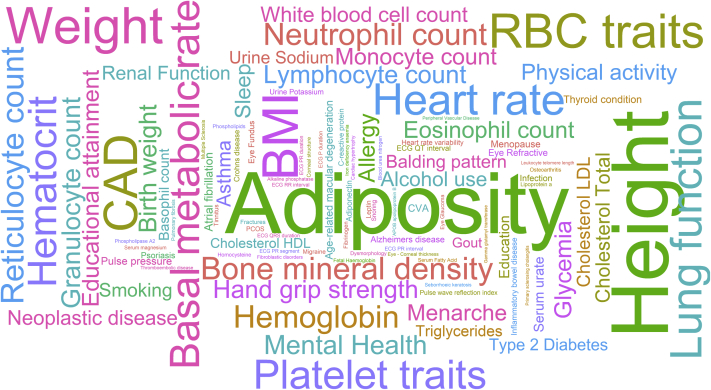
Word cloud generated from all of the genome-wide association studies–identified blood pressure (BP) single-nucleotide polymorphism (SNP) associations with non-BP traits with a *P* value threshold of 58 × 10^−5^. The size of the words indicates the weight based on the number of independent BP SNPs associated with each phenotype.

Pleiotropy occurs when a given genetic locus (SNP) influences 2 or more different phenotypes or traits. Phenome-wide association studies (PheWAS) can identify statistical associations between a single variant and multiple phenotypes and thus reveal pleiotropic associations. PheWAS are usually carried out with the use of a wide range of phenotype data from electronic health records, epidemiologic studies, and clinical trials. Whereas GWAS typically investigates a single phenotype at a time, PheWAS identify all of the traits associated with a genetic variant. With near ubiquitous availability of genome-wide genotyping in most large epidemiologic cohorts and the emergence of biobanks, there is potential to identify pleiotropic SNPs to improve our understanding of the biological functions of a GWAS SNP or identify concealed pathophysiologic connections between traits previously considered as distinct. A look-up of all the 1477 BP SNPs in the GWAS catalogue and PhenoScanner^25^^,^^26^ using a *P* value threshold of 5 × 10^−5^ showed a range of significantly associated traits. The pleiotropic traits are summarised in a word cloud (Fig. 2), where the size of the words in the word cloud indicates the weights based on the number of independent BP SNPs associated with each phenotype. There is considerable overlap of BP SNPs across traits that predispose to or correlate with type 2 diabetes mellitus (T2DM). This suggests that the co-occurrence of T2DM and HTN may manifest through shared genetic factors and shared pathways. However, detecting an independent BP effect on T2DM causation, even if it exists, may be challenging. This is highlighted by 2 recent studies that tried to establish a causal effect of BP on T2DM. One study used 28 SNPs associated with T2DM and showed that a 1 mm Hg rise in genetically determined SBP was causally associated with a 2% increased risk of hypertension.^27^ However, a more recent bidirectional mendelian randomisation study of UK Biobank participants, using a larger SNP set of 134 T2DM SNPs and 233 BP SNPs, suggested that T2DM may causally affect HTN, whereas the relationship from HTN to T2DM is unlikely to be causal.^28^ Nevertheless, genetics provides support for observed correlation between T2DM and HTN and the presence of shared pathways raises the possibility of combined treatment for both. The newer SGLT2 inhibitors with dual effects on both glycemic control and BP are a fortuitous example of a single drug with joint effects on BP and glycemia and support investigation of shared pathways to develop novel therapies for both conditions.^29^

## GWAS and Pharmacogenomics

The goal of genomics is to enable precision medicine through a greater understanding of molecular pathways that regulate BP, which can inform new drug development, personalisation of treatment, and ultimately lead to a new taxonomy of HTN.^4^ But there are significant challenges in realising this goal. Current treatments of HTN have not seen any new drug approval for more than 2 decades, mainly owing to the view that current HTN management is well served by the existing set of drug classes available. Tailoring of therapy has not progressed beyond considering self-reported African ancestry and serum renin levels.^30^^,^^31^

**Figure 3:**
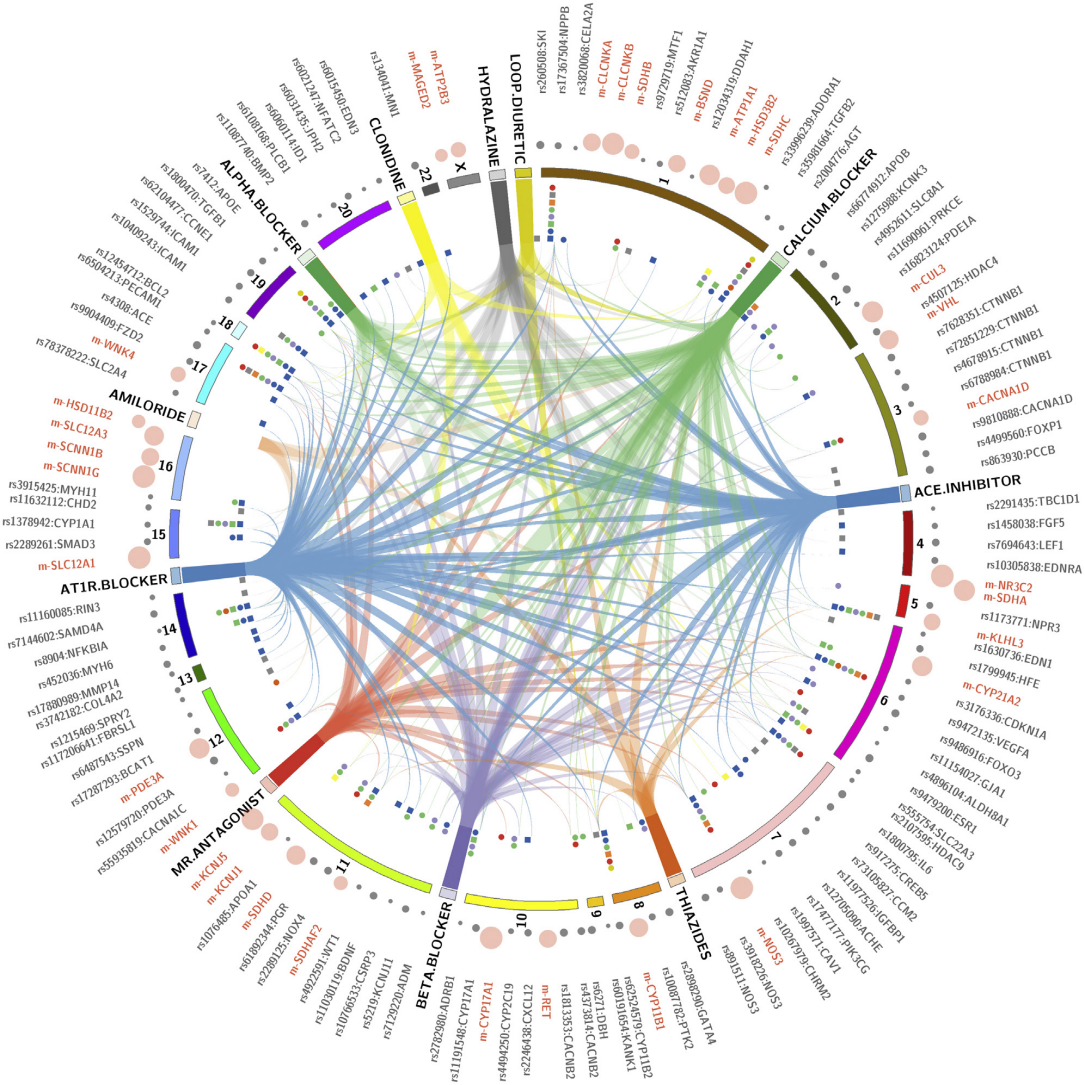
Pharmacogenetic landscape of blood pressure. The Circos^37^ plot shows all the genome-wide association studies–identified blood pressure single-nucleotide polymorphisms (SNPs) and their putatively linked genes that show interaction with licensed antihypertensive drugs. Monogenic genes are presented in **red** and GWAS SNP genes in **dark grey**. Chromosomes are represented as **numbered bands**. The **coloured square and circular markers** indicate the number of antihypertensive drug classes that each gene interacts with. Drug-gene interactions were obtained from the DrugBank^32^ and Comparative Toxicogenomics Database.^33^

Linking all of the GWAS BP variants mapped to genes to the DrugBank^32^ and Comparative Toxicogenomics Database^33^ shows that all of the major antihypertensive drug classes are captured by pharmacogenetic interaction with these GWAS loci (Fig. 3). Although this may simply reflect the fact that the putative published genes mapped to GWAS SNPs were selected for plausible BP effect, this raises the possibility of exploring other genes in the region of GWAS loci for BP-lowering potential. A more attractive approach is to link pharmacogenetic interactions of GWAS signals with pleiotropic PheWAS results, because this may reveal opportunities for drug repurposing and help to define the most appropriate patient populations to benefit from a drug. Table 2 summarises a set of pharmacogenes that interact with GWAS BP genes and have BP lowering as a documented side-effect. We also show the pleiotropic associations of these loci. Some of these drugs have failed trials for other conditions and others have not been trialled for HTN. ET antagonists and riociguat currently licensed for pulmonary HTN show up as pharmacogenetic interactions with GWAS loci for BP (*EDNRA* and *GUCY1A2*, respectively) and are candidates for clinical trials for potential extension of their current indication to essential HTN. Valproic acid shows multiple interactions with a range of GWAS loci (*HDAC9*, *SCN2A*, *SCN10A*), indicating that it may be a candidate for repositioning. Nesiritide interacts with *NPR3* and is currently not licensed because it failed in a heart failure clinical trial. PheWAS results indicate that pharmacogenes for valproic acid are also associated with adiposity traits and heart rate, pharmacogenes for ET antagonists are associated with CAD, and nesiritide pharmacogene is associated with adiposity, basal metabolic rate, height, lung function, and visceral fat traits. These raise the possibility that new drug development or drug repurposing needs to take into account multimorbid associations for targeting treatment to the right subset of patients. Finally, instead of repurposing drugs, pleiotropic associations open the possibility of multipurposing drugs, for example, prioritising antidepressant drugs that additionally lower BP for patients with hypertension and depression or antiepileptics that also have BP-lowering potential for patients who have both epilepsy and hypertension (Table 2).

**Table 2:** Pharmacologically active gene loci from genome-wide association studies (GWAS), their pleiotropic associations, and key drug-gene interactions with their indications and blood pressure (BP) effects

GWAS locus	Pleiotropic associations	Antihypertensive license	BP reduction as side-effect	Effect on BP	Therapeutic context/indication
*ACE*	Nonpleiotropic	Angiotensin-converting enzyme inhibitors		↓↓↓↓	Hypertension; Heart failure; Diabetes mellitus nephropathy
			Omapatrilat	↓↓	(Hypertension; Heart failure—failed because of adverse drug reactions)
*ACVR2A*	Basophils, Eosinophils, Neutrophils, Renal function, Urate		Sotatercept	↓	(Pulmonary hypertension—phase II)
*ADORA1*	Nonpleiotropic		Adenosine	↓↓	Supraventricular tachycardias
			Pentoxifylline	↓↓	Peripheral vascular disease
*ADRB1*	Birth weight		Bethanidine	↓↓	(Sympatholytic)
		β-Blockers		↓↓↓↓	Hypertension; Angina; Arrhythmia; Heart failure
			Dobutamine	↓↓	Inotropic support; Cardiac stress testing
			Amiodarone	↓↓	Arrhythmia
*AKR1A1*	Basophils, Granulocytes, Height, Hematocrit, Hemoglobin, Neutrophils, Platelet traits, RBC traits, Reticulocytes, WBC		Tolrestat	↓	(Diabetes complications—failed trials)
*AKR1B10*	Nonpleiotropic				(Diabetes complications—failed trials)
*BCL2*	Adiposity, BMI, BMR, CAD, Glycemia, Hematocrit, RBC traits, Reticulocytes, T2DM, Visceral.fat, Weight		Docetaxel	↓↓	Solid tumours
*CACNA1C*	Hematocrit, Hemoglobin, RBC traits		Cinnarizine	↓	Ménière disease
		Spironolactone		↓↓↓↓	Hyperaldosteronism; Oedema; Heart failure; Hypertension
			Drotaverine	↓↓	Antispasmodic
			Topiramate	↓	Epilepsy; Migraine
		Calcium channel blockers		↓↓↓↓	Angina; Hypertension
*CACNA1D*	Monogenic, Nonpleiotropic		Cinnarizine	↓	Ménière disease
		Spironolactone		↓↓↓↓	Hyperaldosteronism; Oedema; Heart failure; Hypertension
		Calcium channel blockers		↓↓↓↓	Angina; Hypertension
*CACNB2*	Nonpleiotropic	Spironolactone		↓↓↓↓	Hyperaldosteronism; Oedema; Heart failure; Hypertension
		Calcium channel blockers		↓↓↓↓	Angina; Hypertension
*CHRM2*	Heart rate		Pizotifen	↓↓	Migraine
			Disopyramide	↓↓	Arrhythmia
			Cinnarizine	↓	Ménière’s disease
			Acetylcholine	↓↓	
			Amitriptyline	↓↓	Depression; Neuropathic pain; Migraine
*CSK*	Cholesterol, Granulocytes, Hematocrit, Monocytes, Platelet traits, RBC traits, Renal function		Dasatinib	↓↓	Chronic myeloid leukemia
*CYP11B2*	Height	Spironolactone		↓↓↓↓	Hyperaldosteronism; Oedema; Heart failure; Hypertension
*DBH*	Nonpleiotropic		Ascorbic acid	↓	Scurvy
*DDAH1*	Adiposity		Esomeprazole	↓	Peptic ulcer disease
*EDNRA*	CAD		Ambrisentan	↓↓	Pulmonary hypertension
*ESR1*	Adiposity, Height		Dobutamine	↓↓	Inotropic support; Cardiac stress testing
*FGR*	BMI		Dasatinib	↓↓	Chronic myeloid leukemia
*FRK*	Adiposity, Cholesterol, C-reactive protein, Height, Low-density lipoprotein		Dasatinib	↓↓	Chronic myeloid leukaemia
*GUCY1A2*	CAD		Riociguat	↓↓	Pulmonary hypertension
*HDAC7*	Allergy, Asthma, Platelet traits, Reticulocytes		Belinostat	↓	(T-cell lymphoma)
*HDAC9*	Adiposity, CAD, CVA				
*HDAC9*	Adiposity, CAD, CVA		Valproic acid	↓	Epilepsy; Bipolar disorder; Migraine
*HRH1*	Adiposity, BMD, Neoplasm		Mirtazapine	↓↓	Depression
			Pizotifen	↓↓	5-HT, Muscarinic, H1, Alpha-adrenergic antagonist
			Dimenhydrinate	↓	Vertigo
			Histamine	↓↓	
			Cinnarizine	↓	Ménière’s disease
			Amitriptyline	↓↓	Depression; Neuropathic pain; Migraine
*INSR*	Adiposity, High-density lipoprotein, Height, Triglycerides, Urate, Visceral.fat		Insulin	↓	Diabetes mellitus
*KCNJ11*	Adiposity, BMI, Glycemia, Height, T2DM		Diazoxide	↓↓	Hypoglycemia
*LIMK1*	Lung function		Dabrafenib	↓↓	Melanoma
*MTHFR-NPPB*	CAD, RBC traits, Visceral fat	Carvedilol		↓	Hypertension; Angina; Heart failure
*NPR3*	Adiposity, BMR, Height, Lung function, Visceral fat, Weight		Nesiritide	↓↓	(Heart failure—failed clinical trial)
*PDE10A*	Nonpleiotropic		Dipyridamole	↓↓	Adenosine deaminase and phosphodiesterase Inhibitor
			Papaverine	↓↓	(Antispasmodic)
*PDE1A*	Adiposity, BMR, Weight	Calcium channel blockers		↓↓↓↓	Angina; Hypertension
			Bepridil	↓↓	Angina (withdrawn)
*PDE3A*	CAD, Monogenic		Amrinone	↓	Heart failure
*PDE5A*	Basophils, CAD, Granulocytes, Platelet traits, WBC		Dipyridamole	↓↓	Antiplatelet
			Pentoxifylline	↓↓	Peripheral vascular disease
*SCN10A*	Heart rate, Neoplasm		Tetracaine	↓	Local anaesthetic
			Lidocaine	↓↓	Local anaesthetic; Ventricular arrhythmia
			Valproic acid	↓	Epilepsy; Bipolar disorder; Migraine
			Brivaracetam	↓	Epilepsy
*SCN2A*	Adiposity		Zonisamide	↓↓	Epilepsy
			Tetracaine	↓	Local anaesthetic
			Valproic acid	↓	Epilepsy; Bipolar Disorder; Migraine
			Brivaracetam	↓	Epilepsy
*VEGFA*	Hematocrit, Hemoglobin, RBC traits, Renal function, Urate	Carvedilol		↓↓↓↓	Hypertension; Angina; Heart failure
*YES1*	Nonpleiotropic		Dasatinib	↓↓	Chronic myeloid leukaemia

All pleiotropic associations of GWAS BP single-nucleotide polymorphisms (SNPs) were extracted and categorised into groups of correlated traits. Some SNPs did not show any non-BP associations and were classified as nonpleiotropic. The genes linked to GWAS SNPs were determined by proximity to the SNP and cardiovascular plausibility. Only 1 gene per loci was included. Drug-gene interactions were obtained from the DrugBank and Comparative Toxicogenomics Database, and drug indications were obtained from the British National Formulary and Food and Drug Administration labelled indications.

BMI, body mass index; BMD, bone mineral density; BMR, basal metabolic rate; RBC, red blood cells; CAD, coronary artery disease; CVA, cerebrovascular accident; T2DM, type 2 diabetes mellitus; WBC, white blood cells.

## Polygenic Risk Scores

Because the genetic make-up of an individual is largely stable from birth, genetic information has the potential to act as an early risk predictor. Essential HTN is influenced by multiple genetic variants with small individual effect sizes, so meaningful risk prediction necessitates examining the aggregated impact of these multiple variants. This is through calculation of a polygenic risk score (PRS), which is a mathematical aggregate of risk conferred by all of the SNPs significantly associated with BP. It is important to highlight that the risk information provided by the PRS is different from the risk information from genetic markers of monogenic disorders. The latter is a dichotomous result (either high or low probability of disease), whereas the former provides a wider range of probabilistic risk. In addition, the rare variant genotype points to specific biological impact of the variant, whereas the PRS is an amalgamation of numerous small-effect variants across the genome with no specific pathway implicated. A PRS constructed to use of all of the significant GWAS BP SNPs showed a significant association with stroke, CAD, heart failure, and left ventricular mass, but not for kidney function.^34^ This supports the established association between HTN and cardiovascular outcomes and suggests that progression of renal damage due to HTN may continue despite control of HTN. Although there is considerable interest in the use of PRS as a biomarker for early intervention, currently there is no evidence for the clinical utility of PRS for intervention or disease prevention. It is likely that PRS currently may have limited utility because studies have been conducted in adults over the age of 40 years where disease would already have been established. However, PRS may have more value in identification of younger at-risk individuals, which merits further study. There is limited utility in personalisation of treatment or new drug discovery through PRS, primarily because it is derived from an amalgamation of all genetic variants and do not represent unique pathways.

## Conclusions

Genomic studies have identified the largest set of SNPs for BP compared with other complex traits. A proportion of these may have translational potential, and the challenge is to identify them. Pleiotropic associations may point to novel underlying pathways or potentially subtypes of essential HTN. Early translational application may be through drug repositioning, followed by new drug development. PRSs look attractive, but their clinical utility needs controlled studies, and the potential ethical impacts of their widespread use exacerbating health disparities need further assessment.^35^^,^^36^

## Funding Sources

S.P. is funded by the 10.13039/501100000265Medical Research Council (MR/M016560/1; AIM-HY Study), the 10.13039/501100000274British Heart Foundation (BHF; PG/12/85/29925; CS/16/1/31878), and the BHF Centre of Excellence (RE/18/6/34217).

## Disclosures

The authors have no conflicts of interest to disclose.

## References

[1] Frohlich E.D., Dustan H.P., Bumpus F.M., H Irvine (1991). Page: 1901-1991. The celebration of a leader. Hypertension.

[2] Padmanabhan S., Joe B. (2017). Toward precision medicine for hypertension: a review of genomic, epigenomic, and microbiomic effects on blood pressure in experimental rat models and humans. Physiol Rev.

[3] Funder J.W. (2019). Primary aldosteronism. Hypertension.

[4] Padmanabhan S., Aman A., Dominiczak A.F. (2018). Recent findings in the genetics of blood pressure: how to apply in practice or is a moonshot required?. Curr Hypertens Rep.

[5] Lifton R.P., Dluhy R.G., Powers M. (1992). A chimaeric 11 beta-hydroxylase/aldosterone synthase gene causes glucocorticoid-remediable aldosteronism and human hypertension. Nature.

[6] Williams B., MacDonald T.M., Morant S. (2015). Spironolactone versus placebo, bisoprolol, and doxazosin to determine the optimal treatment for drug-resistant hypertension (PATHWAY-2): a randomised, double-blind, crossover trial. Lancet.

[7] Maass P.G., Aydin A., Luft F.C. (2015). PDE3A mutations cause autosomal dominant hypertension with brachydactyly. Nat Genet.

[8] Spence J.D., Rayner B.L. (2018). Hypertension in blacks: individualized therapy based on renin/aldosterone phenotyping. Hypertension.

[9] Brown M.J. (2012). Platt versus Pickering: what molecular insight to primary hyperaldosteronism tells us about hypertension. JRSM Cardiovasc Dis.

[10] Havlik R.J., Garrison R.J., Feinleib M. (1979). Blood pressure aggregation in families. Am J Epidemiol.

[11] Kupper N., Willemsen G., Riese H. (2005). Heritability of daytime ambulatory blood pressure in an extended twin design. Hypertension.

[12] Niiranen T.J., McCabe E.L., Larson M.G. (2017). Risk for hypertension crosses generations in the community: a multi-generational cohort study. Eur Heart J.

[13] Luft F.C. (2001). Twins in cardiovascular genetic research. Hypertension.

[14] Wellcome Trust Case Control Consortium (2007). Genome-wide association study of 14,000 cases of seven common diseases and 3,000 shared controls. Nature.

[15] Evangelou E., Warren H.R., Mosen-Ansorena D. (2018). Genetic analysis of over 1 million people identifies 535 new loci associated with blood pressure traits. Nat Genet.

[16] Zanchetti A. Essay review. J. D. Swales, editor. Platt versus Pickering: an episode in recent medical history. Med Hist 1986;30:94-96. 10.1017/s0025727300045075 PMC1139585 3511340

[17] Padmanabhan S., Melander O., Johnson T. (2010). Genome-wide association study of blood pressure extremes identifies variant near *UMOD* associated with hypertension. PLoS Genet.

[18] Graham L.A., Padmanabhan S., Fraser N.J. (2014). Validation of uromodulin as a candidate gene for human essential hypertension. Hypertension.

[19] Trudu M., Janas S., Lanzani C. (2013). Common noncoding UMOD gene variants induce salt-sensitive hypertension and kidney damage by increasing uromodulin expression. Nat Med.

[20] Gupta R.M., Hadaya J., Trehan A. (2017). A Genetic variant associated with five vascular diseases is a distal regulator of endothelin-1 gene expression. Cell.

[21] Nikpay M., Goel A., Won H.H. (2015). A comprehensive 1,000 Genomes–based genome-wide association meta-analysis of coronary artery disease. Nat Genet.

[22] Wang X., Musunuru K. (2018). Confirmation of causal rs9349379-*PHACTR1* expression quantitative trait locus in human-induced pluripotent stem cell endothelial cells. Circ Genom Precis Med.

[23] Barton M., Yanagisawa M. (2019). Endothelin: 30 years from discovery to therapy. Hypertension.

[24] Dhaun N., Webb D.J. (2019). Endothelins in cardiovascular biology and therapeutics. Nat Rev Cardiol.

[25] Kamat M.A., Blackshaw J.A., Young R. (2019). PhenoScanner v2: an expanded tool for searching human genotype-phenotype associations. Bioinformatics.

[26] MacArthur J., Bowler E., Cerezo M. (2017). The new NHGRI-EBI catalog of published genome-wide association studies (GWAS Catalog). Nucleic Acids Res.

[27] Aikens R.C., Zhao W., Saleheen D. (2017). Systolic blood pressure and risk of type 2 diabetes: a mendelian randomization study. Diabetes.

[28] Sun D., Zhou T., Heianza Y. (2019). Type 2 diabetes and hypertension. Circ Res.

[29] Zinman B., Wanner C., Lachin J.M. (2015). Empagliflozin, cardiovascular outcomes, and mortality in type 2 diabetes. N Engl J Med.

[30] Whelton P.K., Carey R.M., Aronow W.S. (2018). 2017 ACC/AHA/AAPA/ABC/ACPM/AGS/APhA/ASH/ASPC/NMA/PCNA Guideline for the prevention, detection, evaluation, and management of high blood pressure in adults: executive summary. A report of the American College of Cardiology/American Heart Association Task Force on Clinical Practice Guidelines. Circulation.

[31] Williams B., Mancia G., Spiering W. (2018). 2018 ESC/ESH guidelines for the management of arterial hypertension. Eur Heart J.

[32] Wishart D.S., Feunang Y.D., Guo A.C. (2018). DrugBank 5.0: a major update to the DrugBank database for 2018. Nucleic Acids Res.

[33] Davis A.P., Grondin C.J., Johnson R.J. (2019). The Comparative Toxicogenomics Database: update 2019. Nucleic Acids Res.

[34] Ehret G.B., Munroe P.B., International Consortium for Blood Pressure Genome-Wide Association Studies (2011). Genetic variants in novel pathways influence blood pressure and cardiovascular disease risk. Nature.

[35] Minari J., Brothers K.B., Morrison M. (2018). Tensions in ethics and policy created by national precision medicine programs. Hum Genomics.

[36] Martin A.R., Kanai M., Kamatani Y., Okada Y., Neale B.M., Daly M.J. (2019). Clinical use of current polygenic risk scores may exacerbate health disparities. Nat Genet.

[37] Krzywinski M., Schein J., Birol I. (2009). Circos: an information aesthetic for comparative genomics. Genome Res.

